# Allosteric Modulation of Chemoattractant Receptors

**DOI:** 10.3389/fimmu.2016.00170

**Published:** 2016-05-02

**Authors:** Marcello Allegretti, Maria Candida Cesta, Massimo Locati

**Affiliations:** ^1^Dompé farmaceutici s.p.a, L’Aquila, Italy; ^2^Department of Medical Biotechnologies and Translational Medicine, University of Milan, Segrate, Italy; ^3^Humanitas Clinical and Research Center, Rozzano, Italy

**Keywords:** biased signaling, functional selectivity, chemoattractant, chemokine receptor, leukocyte recruitment

## Abstract

Chemoattractants control selective leukocyte homing *via* interactions with a dedicated family of related G protein-coupled receptor (GPCR). Emerging evidence indicates that the signaling activity of these receptors, as for other GPCR, is influenced by allosteric modulators, which interact with the receptor in a binding site distinct from the binding site of the agonist and modulate the receptor signaling activity in response to the orthosteric ligand. Allosteric modulators have a number of potential advantages over orthosteric agonists/antagonists as therapeutic agents and offer unprecedented opportunities to identify extremely selective drug leads. Here, we resume evidence of allosterism in the context of chemoattractant receptors, discussing in particular its functional impact on functional selectivity and probe/concentration dependence of orthosteric ligands activities.

## Introduction

G protein-coupled receptors (GPCRs) are the largest family of cell-surface receptors encoded by the human genome and are involved in most pathophysiological aspects (1, 2). GPCRs fulfill the vital biological function of transducing effects of extracellular signals (photons, lipids, neurotransmitters, proteins, etc.) across the cellular membrane into the cytosolic space *via* the activation of dedicated signaling pathways. Physiologically, when the extracellular signal interacts with the so-called “orthosteric binding site” of a GPCR, a conformational change occurs that conveys the signal through the plasma membrane, thus triggering intracellular signaling cascades *via* heterotrimeric G proteins and other signal transducers (3). Because of the involvement of GPCRs in a plethora of physiological and pathological processes, this receptor family includes most of the targets of actual and potential drugs (1, 4, 5), thus making GPCRs the largest class of targets for drug discovery.

Selective leukocyte homing *via* chemoattractant/receptor interactions is pivotal for the organization of the immune system and for protection against infectious diseases. Chemoattractants are also key players in the development and exacerbation of immunomediated pathological conditions, such as allergic responses, autoimmune diseases, and other acute and chronic inflammatory disorders, and their fine regulation plays a crucial role for the development of an appropriate immune response (6). Leukocyte chemoattractant ligands include a structurally diverse collection of bioactive molecules, including lipids (leukotrienes, prostaglandins, and platelet-activating factor), peptides (formyl peptides), and proteins (chemokines, non-chemokine cytokines, and defensins). Chemoattractant ligands are recognized by a distinct GPCR family categorized into classical chemoattractant and chemokine GPCRs on the basis of their ligands. Classical chemoattractant GPCRs include formyl peptide receptors (FPR and its variants), the platelet-activating factor receptor (PAFR), activated complement component 5a receptor (C5aR), and leukotriene B4 receptors (LTB4R and its variants). Chemokine GPCRs are subcategorized in four families termed CCR, CXCR, CX3CR, and XCR based on the relative positioning of conserved cysteine residues in the N-terminal domain of their mature ligands. So far, roughly 50 chemokines and at least 18 chemokine GPCRs have been identified in humans (7). Beyond chemokine GPCRs, a group of atypical chemokine receptors (ACKRs), which appear to shape chemokine gradients and dampen inflammation by scavenging chemokines in a G protein-independent β-arrestin-dependent manner, has also recently been recognized (8).

G protein-coupled receptors are integral membrane proteins in constant equilibrium between various functionally distinct conformational states, and this equilibrium is influenced by their exogenous and endogenous ligands (9). Exogenous GPCR ligands can bind to their receptor either competitively (orthosterically) by interacting with the same receptor binding site as the endogenous agonist and are classified as agonists, antagonists, and/or inverse agonists, based on their effects on G protein signaling. Allosteric modulators induce biological responses through interaction with a distinct binding site and can directly modulate binding of orthosteric ligands and their signaling activity. Allosteric modulators have a number of potential advantages over orthosteric agonists/antagonists as therapeutic agents, including greater selectivity for receptor subtypes and the opportunity to identify synthetic ligands for a receptor whose orthosteric binding site has been proven to be chemically intractable, as for glucagon-like peptide 1 receptor agonists (10, 11). However, implications and potentials of allosteric modulation in chemoattractant GPCR biology are far to be fully elucidated, and this review aims at highlighting emerging concepts and open questions.

## Allosterism and GPCR Signaling

The ternary complex model for GPCRs activation, which describes a receptor that moves laterally in the cell membrane to physically couple to a trimeric G protein after activation by an agonist, only accounts for part of the complexity of GPCR-signaling system (12). Ligand binding in the extracellular compartment activates intracellular signals propagated not only through G proteins, but also through β-arrestin and accessory proteins binding, and literature (13) proposes more complex models for receptor activation accounting for multiple signaling states with several conformations stabilized by both different ligands and by single ligand in different conditions. Functional selectivity, probe dependence, and concentration dependence are all properties of chemoattactant receptors’ signaling unraveling aspects of the complex processes underlying receptor activation.

Concentration-dependence signaling accounts for different concentrations of the same ligand inducing different receptor responses (14). The typical bell-shaped dose–response curve of chemoattractant-dependent cell migration represents a clear example of this behavior and is particularly relevant in the biology of chemoattractant receptors as they are sensitive to ligand gradients. As an example, high concentrations of a chemokine ligand, such as CXCL12, have been reported to induce inverse migration of CXCR4 expressing cells in several *in vitro* and *ex vivo* models (15, 16). The biological significance of this phenomenon, defined as chemorepulsion or fugitaxis, has been defined in the specific context of T-cell trafficking during thymic migration (17). A number of explanations have been proposed, including the existence of high- and low-affinity-binding sites for the same ligand and the concentration-dependent dimerization/oligomerization of the cognate receptor (18, 19).

A second property of GPCR signaling is functional selectivity or “biased signaling,” an effect mainly observed for class A and C GPCRs (20), which refers to the ability of different ligands to activate a certain intracellular signaling pathway over another on a given receptor (21). At least three elements contribute to make functional selectivity a key element for chemoattractants: (i) spatiotemporal and tissue-specific expression of chemoattractant receptors and their ligands; (ii) modulation of receptor activity by proteins interacting with the receptor (or receptor oligomers) or making heterocomplexes with the ligand; (iii) receptor-intrinsic biased signaling triggered by different chemokines binding to the same receptor (22). Indeed, several chemoattractant receptors are activated by multiple endogenous ligands, which may activate distinct signaling pathways through the same receptor, thus suggesting the existence of different “active” conformations of the receptor associated with a particular repertoire of intracellular proteins (23). Consistent with this, several examples of chemoattractant receptors with biased signaling have been reported. This is particularly prominent in receptors with a large number of ligands, such as CCR1, which has partial agonists (CCL14, CCL15, and CCL23) becoming fully active after processing of their extended N-terminal domain (24, 25), β-arrestin-biased ligands (CCL5, CC15, and CCL23) (26), and G protein-biased agonists (CCL5) (26). CCR7 is activated by CCL19 and CCL21, which are equivalent for G protein signaling but differ in their GRK and β-arrestin engagement (27, 28), while all CCR2 ligands have balanced G protein/β-arrestin signaling but interestingly CCL8 is biased for signaling to β-arrestin2 vs. β-arrestin1 (29). Most importantly, there is also clear evidence of the biological relevance of biased signaling in chemokine receptors, as the warts, hypogammaglobulinemia, infections, and myelokathexis (WHIM) syndrome is caused by mutations deleting the CXCR4 C-terminal domain which generate receptor variants acting as G protein-biased receptors because compromised in their ability to engage β-arrestin for the absence of relevant phosphorylation residues (30). Finally, in the chemokine field, ACKR represents a striking evidence of β-arrestin-biased signaling receptors (31–33). Thus, not only chemokine receptors are complex signaling molecules able to engage different signaling pathways, but different ligands have biased signaling effects, and this has become of particular relevance considering the chemokine system promiscuity. In this context, the property of allosteric ligands of interacting with ligand-bound receptors introduces a further element of complexity and, not surprisingly, the action of an allosteric modulator may differentially affect receptor functions depending on which agonist is used as activating probe. Probe dependence, a phenomenon widely reported for chemokine receptors (6), is therefore a clear consequence of the cooperativity between orthosteric and allosteric sites. An interesting example of the probe-dependent behavior of allosteric modulators has been reported for a series of CCR1 ligands showing opposite effect on the affinity of two endogenous receptor ligands with not overlapping binding sites. In fact, these metal ion chelating compounds originally selected as full CCR1 agonists were found to act as allosteric enhancers of CCL3 binding while displacing CCL5 binding at the orthosteric site (34). Similarly, AMD3100 acts as a potent allosteric inhibitor of CXCL12-induced CXCR4 activation but does not affect receptor-mediated response to CXCL12 peptide fragments with agonistic properties (35). Allosteric-biased modulation on GPCRs can also occur between G proteins and other signaling effectors, such as β-arrestins, as demonstrated in the case of a CXCR4 allosteric modulator (36). If, on the one hand, probe dependence gives a very high hurdle to the characterization of allosteric ligands; on the other hand, it offers unprecedented opportunities to identify extremely selective drug leads, allowing a fine modulation of receptor-activated signals in complex biological systems.

As discussed, GPCRs are allosteric proteins and G proteins behave as natural endogenous allosteric modulators of this class of receptors. The progressive characterization and identification of functionally conserved allosteric sites in different GPCRs unavoidably raise the question whether these sites may represent binding motifs for unknown ligands, physiologically behaving as allosteric receptor modulators (37). In this perspective, a huge number of natural substances belonging to diverse chemical classes (ions, lipids, and peptides) have been reported as putative endogenous allosteric modulators of GPCRs. Our studies have highlighted the functional relevance of a minor pocket conserved in both classical chemoattractant and chemokine GPCRs accounting for the fine regulation of receptors activation and not involved in the orthosteric ligand binding (38) (see below). The existence of specific endogenous ligands behaving as non-competitive allosteric modulators interacting at this minor pocket represents an attractive work hypothesis.

While offering unprecedented opportunities for the design of highly selective pharmacological tools, the allosterism concept implies a profound revision of the entire drug discovery process having impact on the design and characterization of novel lead candidates targeting the GPCR family. From the structural point of view, biased allosteric modulation, probe dependence, and ligand cooperativity require the ability to model multiple conformational states in the presence of different ligands that still represents a major hurdle for the rational design of drugs. Several independent studies have shown how subtle structural and electronic modifications in a class of allosteric GPCRs modulators may result in dramatic changes of the biological activity, thus limiting the possibilities to conduct large and efficient lead optimization programs (39–41). In this context, the synthesis of focused iterative libraries with limited structural variability is often more efficient than the classical high-throughput screening of large diverse chemical libraries. Furthermore, the biological characterization of a new class necessitates a multistep approach that carefully takes into account the multifaceted characteristics of the allosteric modulation mechanisms. When multiple endogenous ligands for the target receptor are reported, as for chemokine receptors, several *in vitro* assays using different probes are recommended for a correct evaluation of probe-dependent effects. The complexity further increases when biased signal is considered, in fact the development of several functional assays in relevant cellular systems is crucial to assess the effect of selected leads on the different signaling pathways including non G protein-mediated signaling. In many cases, the ideal drug profile for the treatment of a specific pathological condition may be scarcely predicted *a priori*; thus, the ultimate goal of a lead optimization program should be the selection of several chemical classes with distinct *in vitro* (probe dependence and functional selectivity) profiles to be in parallel evaluated in relevant *in vivo* models.

## Negative and Positive Allosterism

**Figure 1:**
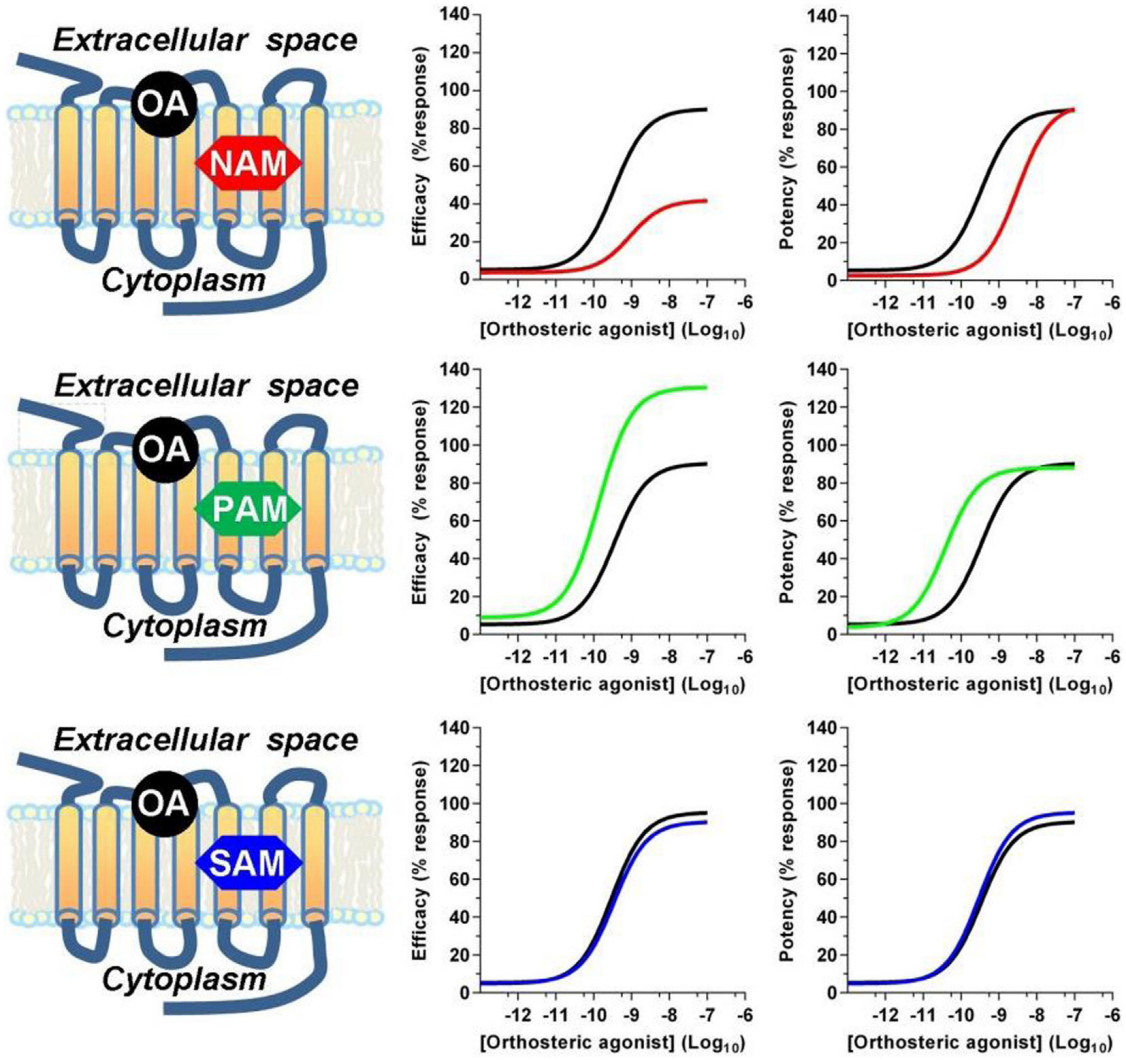
**Allosteric modulators effects on orthosteric agonist efficacy and potency**. Positive (PAM) and negative (NAM) allosteric modulators modulate the affinity and/or the efficacy of orthosteric agonists, while silent allosteric modulators (SAM) have no effect on the affinity and/or efficacy mediated by the orthosteric agonist. Abbreviations used: OA, orthosteric agonist; NAM, negative allosteric modulator; PAM, positive allosteric modulator; SAM, silent allosteric modulator.

Receptor allosteric sites are normally devoted to bind endogenous mineral cations, such as sodium, calcium, zinc, and magnesium, or synthetic drugs (42). From a structural point of view, allosteric modulators can be unrelated to the structure of competitive agonists or antagonists. Within the A class GPCR family, orthosteric binding sites are highly conserved and amino acid sequences necessary for the binding of endogenous ligands are retained, while allosteric modulator binding sites show a great structural diversity, thus displaying a high selectivity for a receptor subtype (43). Allosteric modulators can promote or reduce the binding affinity of orthosteric ligands *via* conformational coupling between the orthosteric and allosteric binding sites, or modulate efficacy by altering the functional response of the receptor to orthosteric ligand binding, thus resulting in positive, negative, or neutral effects on receptor activation (Figure 1). Negative allosteric modulators (NAMs) bind at the allosteric site to inhibit the efficacy or affinity of agonists to the orthosteric site and do not have any intrinsic agonist efficacy. This effect occurs either by stabilizing an inactive conformation of the receptor or by raising the energy barrier requested to activate the receptor (44). NAMs produce rightward and/or downward shifts in agonist concentration–response curves. This can result from the NAM decreasing agonist affinity (at equilibrium) by stabilizing a lower affinity receptor conformation, from the NAM increasing the energy barrier for transition to the active state, or both. The degree of shift is finite and reaches a maximum as the allosteric site is fully occupied by the NAM, differently from what occurs with competitive orthosteric antagonists which produce ever greater shifts at increasing concentrations with no theoretical limit, because of the direct competition for the agonist-binding site (44). Positive allosteric modulators (PAMs) bind to their allosteric site and either promote the binding of the agonists at the orthosteric site or lower the energy barrier necessary to shift the receptor to the active conformation. PAMs do not display any activity or pharmacological effect in the absence of the endo/exogenous agonists, but when combined with an orthosteric agonist, they increase its efficacy, thus improving the overall side-effect profile of the agonist. From the mechanistic perspective, NAMs and PAMs can exert their effects either by altering the binding affinity of the orthosteric ligand or by inducing a conformational change that affects the ability of the ligand/receptor complex to propagate the stimulus to intracellular proteins. Finally, neutral allosteric ligands, previously referred to as silent allosteric modulators (SAMs), have no effect on orthosteric agonists affinity or efficacy but are able to act as competitive antagonists at the same allosteric site and block PAM or NAM activity, and are often used to confirm the receptor engagement by NAMs or PAMs (45).

## Allosteric Modulation of Chemokine Receptors

The most relevant efforts to develop chemokine receptor inhibitors have been focused on drugs blocking HIV infection (see Table 1). This effort led to the registration as anti-HIV drug of the CCR5 antagonist Maraviroc (Celsentri/Selzentry; Pfizer), while the CXCR4 antagonist Plerixafor (Mozobil/AMD-3100; Genzyme), originally developed as a second anti-HIV drug, was subsequently assessed as an hematopoietic stem cell mobilizer and is now indicated in combination with G-CSF to mobilize stem cells to the peripheral blood in autologous transplantation in patients with non-Hodgkin’s lymphoma and multiple myeloma. Conversely, a number of clinical trials of chemokine receptor antagonists for immunomediated diseases have been disappointingly unsuccessful, generally due to a lack of efficacy in Phase II for inappropriate target selection and/or insufficient receptor coverage. However, new clinical programs in focused indications are ongoing, setting the premises for a better understanding of the therapeutic potential of these important targets. Among these, 29 drugs are reported as “allosteric modulators of chemokine receptors,” three of them being in Phase II and two in Phase III of development (Thomson Reuters Cortellis Business Intelligence; https://cortellis.thomsonreuterslifesciences.com).

**Table 1:** **Selected inhibitors of chemokine and chemoattractant receptors**.

Name	Structure	MoA	Company	Stage	Indication
Cinacalcet	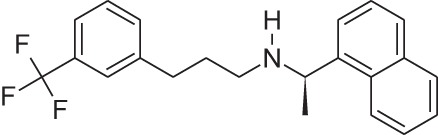	CaSR PAM	Amgen	M	End-stage renal disease
Plerixafor (AMD3100)	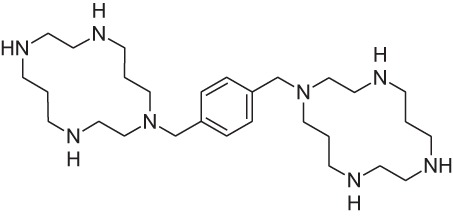	CXCR4 NAM	Genzyme	M	Bone marrow transplantation
Maraviroc	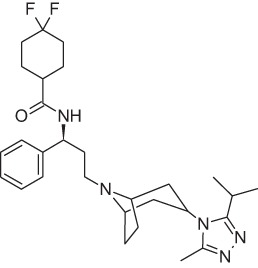	CCR5 NAM	Pfizer	M	HIV
Reparixin (DF1681Y)	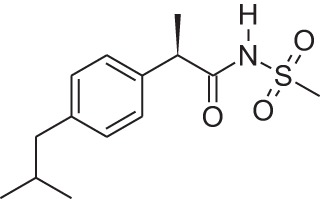	CXCR1 NAM	Dompé farmaceutici	III	β-cell transplantation
Ladarixin (DF2156A)	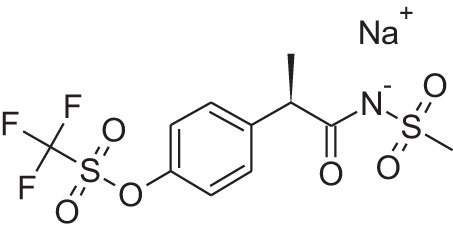	CXCR2 CXCR2 NAM	Dompé farmaceutici	II	Onset type 1 diabetes
Navarixin	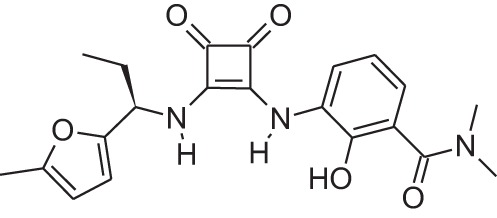	CXCR2 NAM	Pharmacopeia	D	COPD
PMX-53	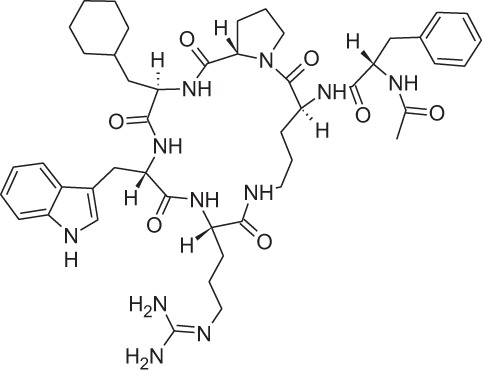	C5aR antagonist	Cephalon (now Arana)	D	Immunity inflammation
Avacopan (CCX-168)	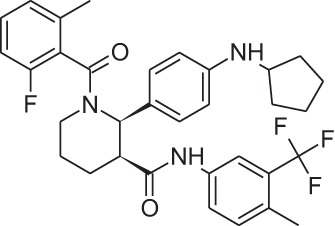	C5aR antagonist	ChemoCentrix	III	ANCA vasculitis
DF2593A	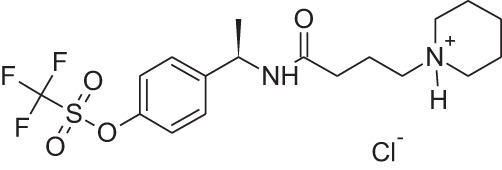	C5aR NAM	Dompé farmaceutici	P	Pain

*CaSR, calcium-sensing receptor; HIV, human immunodeficiency virus; COPD, chronic obstructive pulmonary disease; ANCA, anti-neutrophil cytoplasmic antibodies; M, marketed; P, preclinical; D, discontinued*.

Reparixin (formerly known as repertaxin) and ladarixin represent the first examples of non-competitive allosteric modulators of chemokine receptors, showing the ability to behave as NAMs of CXCR1/CXCR2 without affecting the cognate ligand binding affinity. Interleukin-8 (IL-8; CXCL8) and related ELR^+^ CXC chemokines are able to interact with CXCR1 and CXCR2 at a different degree, with IL-8 and CXCL6 being potent agonists for both CXCR1 and CXCR2, whereas the other chemokines show a higher selectivity degree toward the CXCR2 subtype. CXCR1 and CXCR2 are largely expressed on PMNs but also T lymphocytes and natural killer cells, and play a key role in leukocyte trafficking in inflammatory conditions (46–49). The contribution of IL-8 and its CXCR1/CXCR2 receptors to the physiopathology of several acute and chronic inflammatory conditions, from ischemia/reperfusion injury to chronic obstructive pulmonary disease and fibrosis, is well assessed by the scientific literature (50–53). Modulators of CXCR1 and CXCR2 function may be useful to treat chronic inflammatory conditions in humans (46). Reparixin was the first known non-competitive allosteric inhibitor of IL-8 receptors, with a 400-fold higher efficacy in inhibiting CXCR1 activity than CXCR2. Its molecular mechanism of action was thoroughly investigated showing that the molecule binds CXCR1 in an allosteric site spanning between transmembrane helices (TM) 1, 3, 6, and 7 and inhibits the signaling triggered by IL-8 without affecting its binding to the receptor (54). The efficacy of the molecule in preventing PMN recruitment and associated tissue damage was demonstrated in experimental models of ischemia/reperfusion injury (2, 55, 56) and organ transplantation (57), thus paving the way to clinical development. The molecule recently completed the first Phase III trial aimed at demonstrating its efficacy in the prevention of graft loss in allogeneic pancreatic islet transplantation, thus confirming the validity of the approach. The knowledge of reparixin molecular mechanism of action paved the way to a rational design approach to identify potent dual CXCR1/CXCR2 inhibitors with improved pharmacokinetic properties suitable for long term administration (41). Ladarixin (DF 2156A), the second clinical candidate in this class, is a highly potent CXCR1 and CXCR2 inhibitor (IC_50_ = 0.1 nM) that is able to block in a probe-independent manner the receptor activation process. Interestingly, mechanistic studies support the rationally derived binding mode hypothesis, thus confirming that the allosteric site is conserved among the two receptor subtypes. The binding mode of the molecule with CXCR1 and CXCR2 is in keeping with the concept that allosteric sites in the TM domains of GPCRs could represent valuable targets for selective allosteric inhibitors able to finely modulate receptor signaling, and suggests their therapeutic investigation in inflammatory disorders. Pharmacological studies were conducted to investigate the potency of CXCR1/CXCR2 inhibition for the prevention of inflammation- and autoimmunity-mediated damage of pancreatic islets. Blockade of CXCR1/CXCR2 was associated with inhibition of insulitis and modification of leukocytes distribution in blood, spleen, bone marrow, and lymph nodes, and was effective in preventing diabetes in an inflammation-mediated model based on multiple low dose injections of streptozotocin and in preventing diabetes in NOD mice (58). Pharmacokinetic, toxicological, and pharmacodynamic data have reinforced the therapeutic clinical potential of Ladarixin, and a Phase II clinical study to test ladarixin at the onset of type 1 diabetes has been recently activated with the aim to confirm this strategy and further investigate its potential in preserving residual β-cell function.

## Allosteric Modulation of Classical Chemoattractant Receptors

The complement has long been recognized as a potentially useful therapeutic target, and a number of strategic approaches and therapeutic agents have been developed during the last years (see Table 1) (59, 60). Inhibition of complement activation has been approached with low molecular weight natural and synthetic compounds, polypeptides, and macromolecules; nevertheless, to place in the market, a complement-directed drug resulted more challenging than expected. Eculizumab, a humanized mAb against C5, was approved for the treatment of rare disorders only in 2007, then followed in 2008 by the approval of nanofiltered C1 inhibitor, and by the orphan drug designation for the human mAb OMS721 targeting mannan-binding lectin-associated serine protease-2. C5 and its GPCR C5aR have been the main targets for the inhibition of complement activation, with two molecules that have reached clinical stage. In Phase I clinical trials in rheumatoid arthritis and psoriasis, the C5aR cyclic peptidomimetic antagonist PMX-53 (Cephalon, now Arana) was found safe and well tolerated, and able to block C5aR at a stage in immune and inflammatory processes earlier than other current anti-inflammatory drugs, but has been discontinued in 2012 due to poor pharmacokinetic profile and off-target side effects. CCX-168 (now avacopan) ChemoCentrix is an orally administered C5aR inhibitor under development for various autoimmune disorders, including ANCA-associated vasculitis, atypical hemolytic uremic syndrome, and IgA nephropathy. Recently, positive top-line data from CCX-168 Phase II CLEAR trial have been announced in patients with ANCA-associated vasculitis, paving the way for a Phase III trial announced to start within 2016. At the same time, other Phase II trials in rare and orphan indications are ongoing (Thomson Reuters Cortellis Business Intelligence; https://cortellis.thomsonreuterslifesciences.com).

Among molecules still at a preclinical stage, DF2593A represents an interesting case of study on the topic of GPCR allosteric/regulatory sites. Our studies on C5aR were guided by the hypothesis that a minor allosteric pocket conserved across the TM region of the chemoattractant receptor family could represent a “triggering domain” crucial for the fine tuning of receptor activation (61). This pocket spanning between TM 1, 3, 6, and 7 was the same reported as the binding site of reparixin and ladarixin. Despite the low homology between C5aR, CXCR1, and CXCR2, the key features of the minor pocket were found conserved, thus allowing the rational design of DF2593A as a putative high affinity selective ligand of C5aR. Extensive mutagenesis studies confirmed the mechanistic hypothesis showing that this region, as observed for CXCR1 and CXCR2, is not involved in orthosteric ligand biding but essential for intracellular signal transduction and receptor function (61). DF2593A was shown effective in several animal models of acute and chronic inflammatory and neuropathic pain (61), and is currently under evaluation as a potential clinical candidate in these indications. These studies further confirm the great potential of allosteric modulation as a promising strategy to generate potent and selective modulators of chemoattractant receptors.

## Allosteric Modulators: New Opportunities for Drug Discovery

Studies on allosteric ligands with different binding properties at cognate GPCRs have led to a substantial increase in our understanding of GPCR pharmacology, thus smoothing the way to the design of safer, better-tolerated, and more efficacious drugs. The recent advances in GPCR structure biology, with the elucidation of several high resolution crystal structures of GPCRs [references in Ref. (62)], will give a further significant boost to this complex and stimulating research field (62), offering new tools for the rational design of allosteric modulators.

As thoroughly discussed, allosteric GPCR modulators present unique advantages as compared to orthosteric ligands, mostly by virtue of their high receptor subtype selectivity and functional selectivity. The first characteristic relies on the greater divergence in the amino acid sequence of allosteric sites between receptor subtypes. While functionally conserved, allosteric sites apparently evolved under a lower evolutionary pressure as compared to the orthosteric sites involved in the recognition of the endogenous ligand. The functional selectivity and probe-dependence properties of many allosteric modulators are intrinsically associated with ability of these molecules to fine tune the dynamic conformational rearrangement of the receptor and ligand/receptor complexes. This second level of selectivity may offer unprecedented opportunities for the design of tailored pharmacological tools but also implicates a profound evolution of the drug discovery process demanding for a deep *in vitro* characterization of the new lead candidates for a correct interpretation of *in vivo* studies results. A fascinating aspect of this research field originates from the continuous mutual feedback between drug discovery and receptor biology research: in a virtuous circle, new lead candidates stemmed from medicinal chemistry programs become important research tools useful to improve the knowledge of GPCR structure and function that fundamentally influence the drug design and development process.

From the drug discovery point of view, allosteric modulators may provide functional advantages over classical orthosteric agonists and antagonists (63). First, as allosteric modulators do not compete with endogenous ligands, their effect on GPCRs is saturable, meaning that when all allosteric sites are occupied no more effects are achieved. Second, as allosteric ligands modulate activities of endogenous ligands engaging the orthosteric site, their influence on receptors’ conformation and signaling will be evident only when the endogenous ligand is present. Third, NAMs often show only partial antagonist activity without exhibiting any agonist activity (64), thus suggesting that a partial NAM could have a greater safety index than a full antagonist. Also for PAMs, the above described ligand-dependent activity may improve safety profile, due to the fact that normal physiological regulation of signaling, including temporal regulation, remains unchanged (65).

The multifactorial interactions implicated in chemoattractant biology make very difficult to predict the *in vivo* behavior of allosteric ligands in pathological conditions, and a deep *in vitro* characterization in different conditions is absolutely required for the interpretation of the results of pharmacological studies. Antagonist affinities can vary depending on the agonist, the presence or absence of allosteric ligands, the specific site through which the effect is exerted, and the specific signaling under consideration. Since most GPCRs can engage different downstream signaling pathways, which are often cell-, tissue-, and/or context-specific, it is crucial to take into account the entire signaling repertoire for the drugs of reference in normal vs. pathological conditions. Future research efforts should be oriented toward the development of approaches aiming to elucidate the full spectrum of ligand signaling in different cell models and able to integrate new screening and quantitative analytical methods, with the aim to link these signaling signatures to preclinical or clinical data.

## Conclusion

The discovery of allosteric modulators has represented a profound advance in the research of drugs acting on chemoattractant receptors, and over the last years, several NAMs and PAMs of both chemoattractant receptors belong to both classical chemoattractant (Cinacalcet, PAM of CaSR of PTH) and chemokine GPCRs (Reparixin, NAM of CXCR1; Ladarixin NAM of CXCR1/CXCR2; and Navarixin, NAM of CXCR2) entered clinical trials, and in some cases, successfully reached the market (Maraviroc, NAM of CCR5; Plerixafor, NAM of CXCR4). The pharmacological implications potentially deriving from the availability of multiplicity of molecules that, acting through a single receptor, differentially regulate its signaling activity are still far from being fully exploited and will offer opportunities for the development of new drugs targeting chemoattractant receptors in the near future.

## Author Contributions

MA, MC, and ML contributed equally to writing and critically revised the paper.

## Conflict of Interest Statement

The authors declare that the research was conducted in the absence of any commercial or financial relationships that could be construed as a potential conflict of interest.

## Abbreviations

ACKRs, atypical chemokine receptors; ANCA, anti-neutrophil cytoplasmic antibodies; C5aR, activated complement component 5a receptor; FPR, formyl peptide receptor; GPCRs, G protein-coupled receptors; LTB4R, leukotriene B4 receptor; NAMs, negative allosteric modulators; NOD, non-obese diabetic; PAFR, platelet-activating factor receptor; PAMs, positive allosteric modulators; SAMs, silent allosteric modulators; TM, transmembrane helices; WHIM, warts, hypogammaglobulinemia, infections, and myelokathexis.
